# A Pilot Study to Evaluate the Minimally Invasive Burn Care for Small, Deep Partial-Thickness Burns of the Hands and Feet Using Enzyme Debridement and Autologous Skin Cell Spray

**DOI:** 10.3390/jcm13247721

**Published:** 2024-12-18

**Authors:** Kohei Aoki, Takako Komiya, Kento Yamashita, Kazuki Shimada, Miki Fujii, Hajime Matsumura

**Affiliations:** Department of Plastic and Reconstructive Surgery, Tokyo Medical University, Tokyo 160-0023, Japan; k-aoki@tokyo-med.ac.jp (K.A.); takapinsmile@gmail.com (T.K.); kento0314cruo0@yahoo.co.jp (K.Y.); kazuki7shimada@gmail.com (K.S.); mikidtma@gmail.com (M.F.)

**Keywords:** deep partial-thickness burn, enzymatic debridement, NexoBrid^®^, autologous skin cell spray, ReCell^®^, hand, foot

## Abstract

**Background/Objectives**: We treated deep partial-thickness burns of the hands and feet in four cases using a combination of NexoBrid and ReCell autologous cell regeneration techniques, without conventional split-thickness skin graft, with good results following debridement of the eschar. **Methods**: We report cases of patients treated with a combination of the NexoBrid and ReCell techniques between 1 August 2023 and 31 July 2024. The degree of debridement and the time to complete wound closure were evaluated. Scar quality was assessed using the Vancouver Scar Scale (VSS). **Results**: Four patients aged 0–28 years with an average total burn surface area of 1.2% were treated on two hands and two feet, with an average follow-up of 12 months; no additional surgical treatment was needed. The mean VSS score was 0.25. The patients were satisfied with the aesthetic appearance of their hands and feet, and no complications, such as hypertrophic scars, were observed. We also developed separate algorithms for sedation and analgesia management for adults and children. **Conclusions**: Using ReCell alone following debridement of small burn wounds with NexoBrid resulted in early wound closure with good scar condition and cosmetic appearance.

## 1. Introduction

Surgical excision of the burn eschar and subsequent wound coverage with an autologous skin graft for deep burns are considered the standard of care (SOC). While tangential excision inevitably results in excision of viable tissue, enzymatic debridement with NexoBrid^®^ (Medi Wound, Yavne, Israel) selectively removes nonviable tissue, leaving sufficient viable dermis on the wound surface [1,2,3,4,5,6,7,8,9,10,11,12,13,14,15,16,17,18]. Burned hands and feet are especially challenging owing to their delicate, crowded anatomy, thin skin, minimal subdermal tissue, and functional and cosmetic importance [2,3]. NexoBrid is appropriate for all burn wounds, regardless of the depth. Therefore, it is practical and effective for debriding mixed- or indeterminate-depth burns and scalds [4,5].

Grafts by the application of an autologous skin cell suspension (ReCell^®^, Avita Medical, Inc., Valencia, CA, USA) have been shown to produce early epithelialization when used alone on deep partial thickness (DPT) burns and skin grafts [18,19,20]. When autologous skin cell spray using ReCell system, ReCell technique, are used alone on a DPT burn, early epidermalization, similar to that in conventional skin grafting, has been observed, which allows for early epithelialization and scar reduction with a very small amount of harvested skin, without scar healing of the DPT burn [21].

Full-thickness (FT) burns are not recommended as a stand-alone procedure and should be used in conjunction with autologous stratified reticular grafts to achieve early epithelialization between the reticular meshwork [22].

In this study, we propose a new treatment for DPT burns of the hands and feet using a combination of NexoBrid and ReCell.

## 2. Materials and Methods

### 2.1. Cases

The cases included in this study are retrospective clinical reports of patients treated with NexoBrid combined with ReCell, between 1 August 2023 to 31 July 2024.

**Figure 1:**
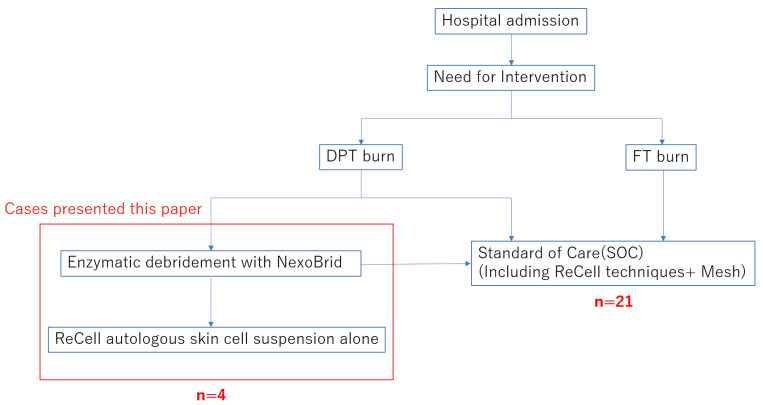
The inclusion criteria for the study.

Each patient with a DPT burn on the hands and feet, assessed as requiring surgery, was treated with NexoBrid and ReCell alone. The inclusion criteria for the study were as follows: (Figure 1)

- DPT burns of the limbs caused by fire/flame, steam, scald, or contact
- Burn wound requiring surgical eschar removal/escharotomy
- Consent for treatment with NexoBrid and ReCell
- The following patients were excluded from the study:
- Cases of FT burn diagnosed after NexoBrid application
- ReCell techniques and mesh skin grafts
- Pregnancy or nursing
- History of allergy and/or known sensitivity to pineapples, papaya, bromelain, or papain
- Pre-enrolment dressings with silver nitrate

Exclusion criteria were listed as

- cases of FT burn diagnosed
- cases treated with SOC
- cases treated with NexoBrid alone
- cases treated with ReCell techniques and mesh skin grafts
- pregnancy or nursing
- history of allergy and/or known sensitivity to pineapples, papaya, bromelain or papain
- pre-enrolment dressings with silver nitrate

Burn depths were assessed following admission according to standard clinical characteristics (color, capillary refill, skin pliability, sensation, presence of blisters, and presence of thrombosed vessels) using video microscopy.

Total burn surface area (TBSA) was determined using a standard Lund Browder chart.

Between August 2023 and July 2024, we treated four patients using the NexoBrid and ReCell techniques. Postoperative follow-up visits were conducted until approximately 1 year.

### 2.2. Enzymatic Debridement (NexoBrid)

Prior to debridement, all patients received routine treatment that included adequate analgesia, as is customary when performing extensive burn dressing. T The wounds were cleansed, blisters were removed, and the burned areas were soaked with saline and 0.05% chlorhexidine for a minimum of 6 h. At this point, the burn wound was covered with gauze and bandages soaked in 0.05% chlorhexidine. In addition, a plastic bag was used to cover the wound to prevent the evaporation of 0.05% chlorhexidine. (In Case 2, the Eco-Probe cover was used instead of a plastic bag to prevent the evaporation of 0.05% chlorhexidine.) As the wound was soaked for a long time, 50–100 mL of saline or 0.05% chlorhexidine was added every 3 h to ensure that the burn wound was constantly soaked. Following the administration of analgesia, NexoBrid was topically applied to the burns wound covered with polyurethane film dressing for 4 h at the patient’s bedside. NexoBrid has a liquid consistency, we firmly attach the film to the burn wound to prevent NexoBrid from leaking. In the case of adults, the burn wound is placed on the top side. In the case of children, the burn wound is placed on the top side under general anesthesia, so they can remain calm. In the case of burns on the dorsum of the foot, a bath towel is placed under the knee to ensure that the entire dorsum of the foot is covered with NexoBrid, taking into account the effects of gravity. In addition, carefully observe whether NxeoBrid is leaking out from the four sides of the film. The treated area was scraped with a sterile tongue depressor to remove the dissolved eschar and Nexobrid remnants. This was followed by a 2-h soaking period using the same solution used prior to debridement. After that, in preparation for the next transplantation with Autologous Cell Suspension, the burn wound surface is covered with an appropriate amount of Vaseline and non-adherent gauze to avoid wound maceration.

### 2.3. Transplantation with Autologous Skin Cell Suspension

DPT skin defects requiring permanent coverage were covered with autologous skin cell suspension grafts at one or two days after enzymatic debridement, considering the patient’s condition and hospital resources. Adults were given a local anesthetic, while children were given a general anesthetic. In the case of adults, skin is taken from the surrounding area, and in the case of children, skin was taken from the hair-bearing area behind the ear, with a thickness of 6/1000 of an inch. After skin grafting using the ReCell technique alone, a layer of SI-Mesh^®^ (ALCARE, Tokyo, Japan) and an adaptive dressing (3M-KCI) is applied, and then covered with gauze. The dressing was applied to donor site in the same way. After that, only the top layer of gauze is changed every two days after the ReCell technique. The burn wound was checked one week after the operation, and after that, the dressing was changed back to vaseline gauze, which is the standard dressing for burns in our institution.

### 2.4. Evaluation and Observation

Following discharge, the treated wounds were assessed weekly for wound closure (percentage of wound surface epithelialized), until complete wound closure (>95% of the wound surface) was achieved.

Photographs of the treated wounds were taken following debridement and autologous skin cell suspension grafting (if performed) at hospital discharge and during weekly and monthly visits.

Several endpoints were selected as measures of the combination efficacy of NexoBrid and ReCell.

- The percentage of DPT wounds closed by autologous skin cell suspension grafting (as a measure of NexoBrid selectivity; assuming selective debridement would spare the viable dermis, which would have the potential for spontaneous epithelialization with less need for autologous skin grafting) and grafted area (as a % of the TBSA).
- Degree of eschar removal (debridement).
- Time to complete wound closure.

Scar quality was assessed using Vancouver Scar Scale (VSS) scores, including scar vascularity, pigmentation, pliability, height, and texture (Table 1).

## 3. Results

Four patients (aged 0–28 years) with an average TBSA of 1.2 were treated with NexoBrid and ReCell techniques alone for burns on the hands and feet, and 21 cases were treated with other treatment modalities (including SOC; standard of care, NexoBrid alone, ReCell techniques alone, NexoBrid and ReCell techniques with mesh graft, etc). The data are presented in Table 2.

**Figure 2:**
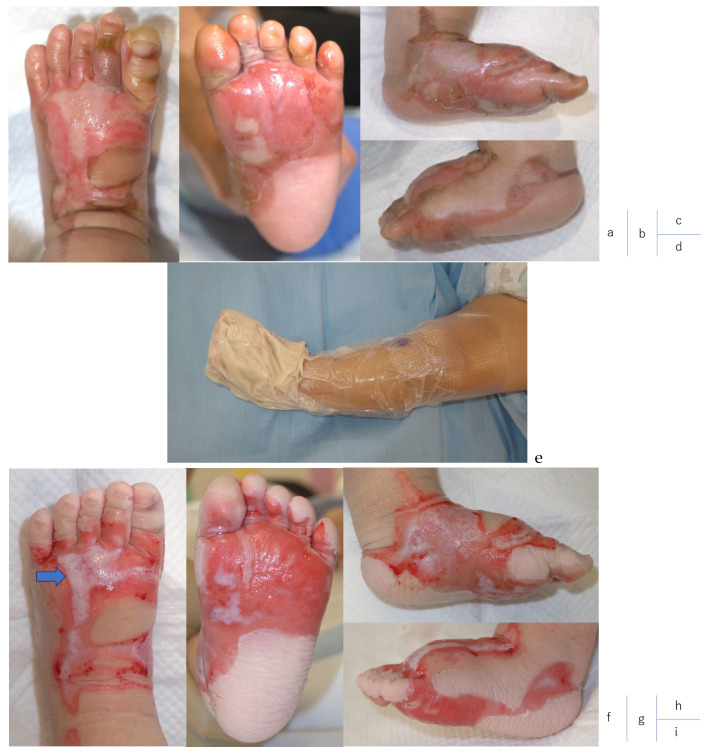

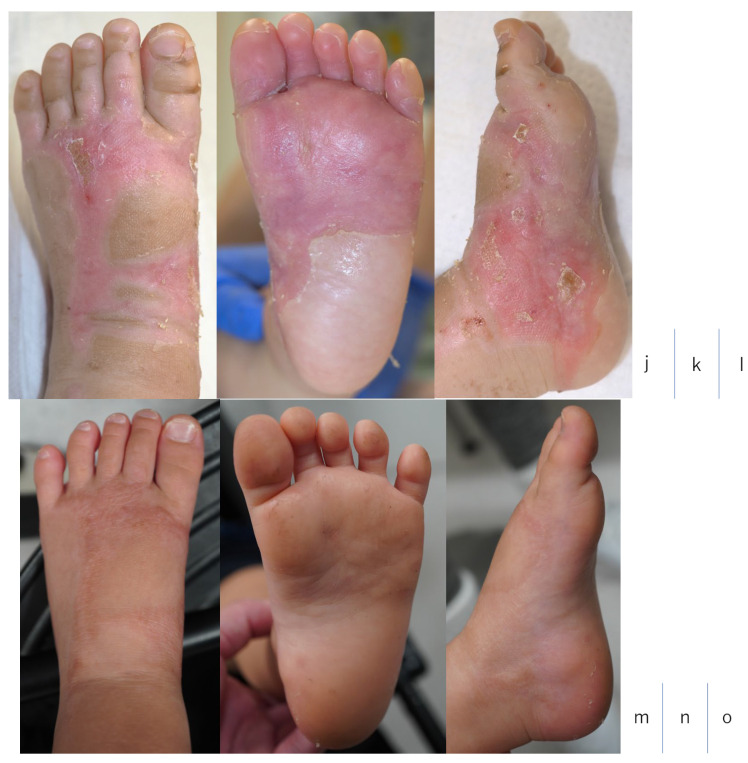
Case 2. (**a**–**d**) Findings on admission. DPT burn; TBSA 2%; caused by scald burn. (**e**) The Echo probe cover was used to immerse NexoBrid. (**f**–**i**) Following debridement with NexoBrid, good petechial hemorrhage was observed in 90% of the total area. Part of the dorsum of the foot (➡; 10% of total area) was inadequately debrided and was debrided with Versaget^®^ (Smith & Nephew, Hull, UK). (**j**–**l**) Ten days following ReCell techniques, the skin graft site was first opened. Epithelialization was observed. (**m**–**o**) One year following NexoBrid and ReCell application. Scars are in good condition, especially on the soles of the feet, and are completely unrecognizable.

**Figure 3:**
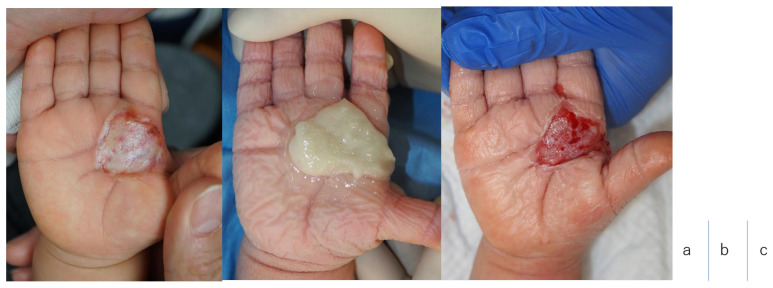

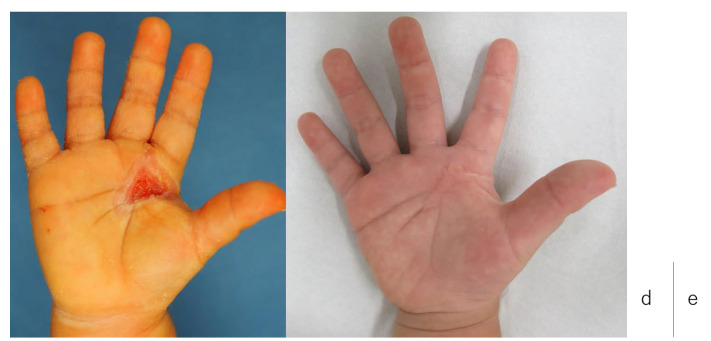
Case 4. (**a**) Findings on admission. The burn was DPT; TBSA 0.2%; caused by a steam burn. (**b**) Debridement with NexoBrid. (**c**) Following debridement with NexoBrid. (**d**) Eleven days following ReCell application, the skin graft site was first opened. The central part of the wound surface did not show epithelialization. All epithelialization was observed 14 days following ReCell application. (**e**) Ten months following NexoBrid and ReCell techniques. The scars are in good condition. There is no restriction on finger extension.

All patients completed the treatment, and they were followed up for 13, 13, 12, and 10 months (Figure 2 and Figure 3) [23]. The patient outcomes were documented (Table 3).

### 3.1. Efficiency of NexoBrid

Most of the eschar was adequately debrided; however, some areas within the burn wounds were not. Case 2 was a DPT burn of the dorsal and plantar surfaces of the foot, but the wound surface was bleeding heavily after NexoBrid application, which was expected to reduce the debridement effect of NexoBrid. Therefore, a portion of the dorsal foot (approximately 10% of the burn area) was deemed inadequately debrided and further debrided with Versajet^®^ (Smith & Nephew) when the skin was grafted with ReCell techniques. In all other cases, except for Case 2, debridement with an Nexobrid was complete. In Case 2, we performed surgical debridement at the site of 10% to completely remove the burn eschar.

### 3.2. Efficiency of ReCell Alone

In all cases, early epithelialization was confirmed using ReCell alone. In Case 1, ReCell-alone techniques were used on an adult on a burn wound on the palmar of the hand, with skin taken from the hypothenar to obtain early epithelialization. All other patients were pediatric, and all skin grafts were obtained from the hairline behind the auricle. The advantage of using ReCell techniques from the hairline was that scarring was not noticeable following epithelialization.

### 3.3. Scar Evaluation

Scar quality was evaluated using the VSS. VSS scores were 0 in Case 1, 0 in Case 2, 0 in Case 3, and 1 in Case 4. The mean VSS score of the entire population was 0.25. Only case 4 was rated 1 for pliabilty. This is because the skin can be easily pinched, but the hardness of the scar is somewhat palpable, so it was rated 1.

In all cases, no complications, such as hypertrophic scars, were observed, and the patients were satisfied with the aesthetic appearance of their hands and feet. No limb flexion or extension impairment was observed after an average of 12 months.

Case 3 in Supplementary file.

## 4. Discussion

NexoBrid was effective against anatomically significant and delicate limb burns with thin skin and little subcutaneous tissue and in the debridement of necrotic tissues, such as DPT burns. The package insert of NexoBrid states that pre-soaking should be done for 2 h, and many facilities do it with this time, but at our institution, we do pre-soaking from the day before and leave it overnight, We are hoping that the burn wound will become more macerated, allowing the enzyme action of NeoBrid to take full effect. In addition, although the European Consensus recommends that the skin grafting procedure be performed 2 days following NexoBrid application [1], we performed ReCell-alone techniques the day after (approximately 12 h later), except in Case 1, where no problems with skin grafting were observed (Case 1 was treated with ReCell techniques 2 days following NexoBrid application). This may be because, after NexoBrid removal, the skin was washed with a large amount of saline solution and thoroughly scrubbed to ensure no NexoBrid residue remained. This was made possible by pain management performed under axillary block in Case 1 and under general anesthesia in the other cases. If the NexoBrid removal procedure is performed in a pain-free state, NexoBrid can be removed without hesitation. Furthermore, as the infant was under general anesthesia, which prevented him from becoming restless, the procedure was performed smoothly, and wound management was simple.

The use of ReCell alone after debridement of small burn wounds with NexoBrid resulted in early wound closure, good scar condition, and good cosmetic appearance. The hair grew back from the hairy skin donor site, of course. It was so much that the surgeon couldn’t even point out the area where the skin had been taken. Conservative treatment may be the treatment of choice for small DPT burns; however, considering that the use of ReCell techniques has resulted in such good cosmetic results (where even the distal palmar cutaneous line can be reproduced), we recommend the aggressive use of such techniques [18]. However, several sweat glands in the palmar and plantar regions easily form a moist environment owing to perspiration when exposed to pain, temperature, or mental strain. Therefore, postoperative pain, room temperature, and mental health control are important factors. In addition, ReCell techniques do not cause depigmentation, as observed in autologous cultured epidermal transplantation. This is because the autologous cultured epidermis contains only epidermal cells, whereas ReCell formulations contain not only epidermal cells but also pigment cells and fibroblasts around the basement membrane. The presence of pigment cells reproduces the natural color tone of the skin [24,25,26]. In all the cases in this study, no depigmentation or hyperpigmentation was observed, and the cosmetic appearance was highly satisfactory.

DPT burn scars develop into complex scars with a mixture of hypertrophic scarring and keloid tissue due to inflammatory cell migration, with a secondary process of wound site shrinkage due to condensation of surrounding cells over time (classification by contour) [27,28,29,30]. This classification is important in determining the correct treatment for burn scars and selecting an appropriate scar assessment method to evaluate the course and efficacy of the treatment itself. The currently used scar assessment instruments include the VSS, Visual Analog Scale, Patient and Observer Scar Assessment Scale, Stony Book Scar Evaluation Scale, and Manchester Scar Scale [31]. The VSS evaluates vascularity, pigmentation, pliability, and height [32] and uses a range of 0–13 points, with lower scores indicating better outcomes. In this case study, we used VSS, a simple and objective assessment tool effective for post-surgical scars, and in this case, it is especially useful as the children are approximately 1 year old and cannot provide a subjective evaluation. The mean VSS score of the entire population was 0.25. The results of this study were very satisfactory, as evaluated using the VSS. In all four cases, no complications were observed, such as hypertrophic scars, and the patients were satisfied with the aesthetic appearance of their hands and feet. DPT burn treatment with a combination of NexoBrid and ReCell alone yielded good results in terms of movement disorders and aesthetics. Adults have almost normal perception, and children have normal objective perception and do not get hurt, so they probably have sufficient protective sensation. To the best of our knowledge, there are no reports on the combined use of NexoBrid and ReCell for DPT burns outside our institution. Therefore, our study contributes novel insights into treatment strategies in such cases. While the results are encouraging, this study should be viewed as a pilot that requires substantial additional research before the technique can be recommended as a standard treatment approach for deep partial-thickness burns.

## 5. Conclusions

Minimally invasive care for DPT burns of the hands and feet using both NexoBrid and ReCell techniques provided efficient skin debridement and quick epithelialization without split skin grafting and resulted in improved functionality and aesthetics.

## Figures and Tables

**Table 1:** Components of the Vancouver Scar Scale.

	Scar Category	Scar Category
Vascularity	Normal	0
	Pink	1
	Red	2
	Purple	3
Pigmentation	Normal	0
	Hypopigmentation	1
	Hyperpigmentation	2
Pliability	Normal	0
	Pliability	1
	Yielding	2
	Firm	3
	Ropes	4
	Contracture	5
Height (mm)	Flat	0
	<2	1
	2~5	2
	>5	3
Best outcomes (lowest score)		0 (13)

A lower score denotes a better outcome when using the Vancouver Scar Scale (range: 0–13).

**Table 2:** Demographics and burn characteristics of patients.

Total Number of Patients Enrolled		4			
		Case 1	Case 2	Case 3	Case 4
Gender		male	male	male	male
Age (years)		28	0	1	1
Depth (burn)		DPT	DPT	DPT	DPT
TBSA (%)		0.5	2	2	0.2
Caused by		contact	scald	scald	steam
Part		palmar	dorsal and plantar	dorsal and plantar	palmar
Pain management	Nexobrid (when applied topically)	Axially nerve block	General anesthesia	General anesthesia	General anesthesia
	ReCell techniques (Including skin taken)	Local anesthesia	General anesthesia	General anesthesia	General anesthesia

**Table 3:** Outcomes of NexoBrid and ReCell application.

	Case 1	Case 2	Case 3	Case 4
Days of initial Nexbrid (days after admission)	1	1	3	3
Efficiency of debridement (%)	100	90	100	100
		yes		
Surgical debriedment required after Nexobrid	no	(Only 10%	no	no
		using Versajet)		
Additional treatments needed after ReCell techniques	no	no	no	no
Days to comlete healing burn wound after ReCell techniques’	10	10	8	14
Days to complete healing skin donor site wound after ReCell techniques	10	6	8	8
Vancouver Scar Scale (VSS) scores				
Vascularity	0	0	0	0
Pigmentation	0	0	0	0
Pliability	0	0	0	1
Height	0	0	0	0
[SUM]	0	0	0	1
Follow-up investigation (months)	13	13	12	10

The burn wound was opened 10 days following ReCell application and may have epithelialized within 10 days in Cases 1 and 2. The skin donor site was also opened 10 days following ReCell application and may have epithelialized before 10 days in Case 1. Epithelialization is defined as 90% or more epithelialization per total area.

## Data Availability

All data analyzed in this study are available from the corresponding author upon request.

## Supplementary Materials

The following supporting information can be downloaded from https://www.mdpi.com/article/10.3390/jcm13247721/s1, and Case 3 photos are shown. Photographs of Case 1 can be found in Reference [18].
