# Supplementary material for: A Pilot Study to Evaluate the Minimally Invasive Burn Care for Small, Deep Partial-Thickness Burns of the Hands and Feet Using Enzyme Debridement and Autologous Skin Cell Spray
Source: J Clin Med. 2024 Dec 18;13(24):7721. doi: 10.3390/jcm13247721 (PMC11678635; doi:10.3390/jcm13247721)
Supplement: Supplementary file 1 [file jcm-13-07721-s001.zip › jcm-3364024-supplementary.pdf]

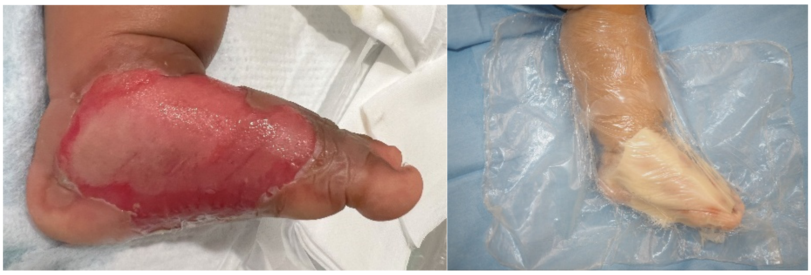

a | b

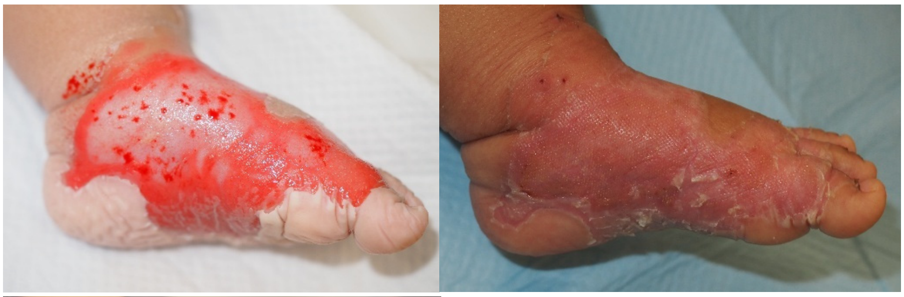

c | d

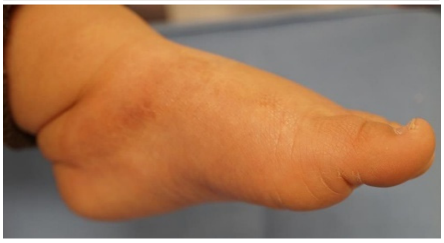

e

Supplementary file. Case 3. (a) Findings on admission. The burn was DPT; TBSA 2%; caused by a scald burn. (b) Debridement with NexoBrid. (c) Following debridement with NexoBrid. (d) eight days following ReCell application, the skin graft site was first opened. All epithelialization was observed. (e) Ten months following NexoBrid and ReCell techniques. The scars are in good condition. There is no restriction.
